# How We Might Further Integrate Considerations of Environmental Impact When Assessing the Value of Health Technologies

**DOI:** 10.3390/ijerph191912017

**Published:** 2022-09-22

**Authors:** Barbara Greenwood Dufour, Laura Weeks, Gino De Angelis, Dave K. Marchand, David Kaunelis, Melissa Severn, Melissa Walter, Nicole Mittmann

**Affiliations:** Canadian Agency for Drugs and Technologies in Health, Ottawa, ON K1S 5S8, Canada

**Keywords:** environment, health technology assessment, Canada, health systems, climate change, deliberative process, health care, health policy, sustainability, guideline

## Abstract

There is growing awareness of the impact health technologies can have on the environment and the negative consequences of these environmental impacts on human health. However, health system decision-makers may lack the expertise, data, or resources to incorporate environmental considerations when making decisions about the adoption and use of health technologies. In this article, we describe how health technology assessment (HTA) is evolving to address climate change by providing health system decision-makers with the information they can use to reduce the impact of health care systems on the environment. Our objective is to consider approaches for including the environment domain when conducting an HTA—in particular, the use of the deliberative process—and for determining when the domain should be included. We explore the challenges of gathering the relevant data necessary to assess the environmental impact of a health technology, and we describe a “triage” approach for determining when an in-depth environmental impact assessment is warranted. We also summarize related initiatives from HTA agencies around the world.

## 1. Introduction

There is growing awareness of the impact health technologies can have on the environment and the negative consequences of these environmental impacts on human health [1,2]. The greenhouse gas (GHG) emissions of the health care sector are commonly cited as an example of the impact of health care on the environment. This is for good reason—if health care were a country, it would rank 5th in GHG emissions [3]. In Canada, GHG emissions are estimated to be responsible for 373 to 581,000 disability-adjusted life-years’ worth of health damages [4]. Pharmaceutical production alone accounts for 25% of GHG emissions from health care in Canada [2].

However, health care contributes to climate change in other ways as well. Health care activities produce a high amount of waste, including disposable gloves, bandages, masks, syringes, catheters, etc., and the materials these products are packaged in. This waste, along with other waste humans produce, is overfilling our landfills and ending up in oceans and waterways [5]. It could be argued that not enough effort is being spent on addressing this issue by reducing the overuse of these products, by using reusable products, or by using less packaging.

Health care is also a major contributor of toxic chemicals to the environment. The toxic chemicals in health care waste seeps into the environment and, during the manufacture and use of health technologies, contaminants are released into the air, water, and soil. These chemicals, which include endocrine-disrupting chemicals and heavy metals, are known to have serious impacts on human health and the environment [5].

The environmental impacts of health care often affect Indigenous and marginalized communities to a greater extent, and climate change will further widen existing health care gaps [6,7].

Health technology assessments (HTA) provide health policy decision-makers with information on the value of health technologies that they can use when making funding decisions. HTA “is a multidisciplinary process that uses explicit methods to determine the value of a health technology at different points in its life cycle. The purpose is to inform decision-making in order to promote an equitable, efficient, and high-quality health system [8]”. Assessments of health technologies, which can be tests, devices, medicines, vaccines, procedures, programs, or systems [9], typically look at a technology’s clinical effectiveness, cost-effectiveness, and safety. However, assessments can include evidence and perspectives from several other domains—ethical, social, cultural and legal issues; the wider implications of the technology to patients, their relatives and caregivers, and the population; and the potential effects of the technology on the environment [8].

The governments of countries around the world have committed to reducing the contribution of health care to climate change [10]. However, health system decision-makers may lack the expertise, data, or resources to incorporate environmental considerations when making decisions about the adoption and use of health technologies. Therefore, there is increasing interest among HTA agencies to more frequently or consistently incorporate environmental considerations into assessments of health technologies [11].

Our objective is to consider approaches for including the environment domain when conducting an HTA and for determining when the domain should be included. We also aim to summarize related initiatives from HTA agencies around the world. The opinions we express are informed by previous and ongoing work that CADTH has undertaken on this topic. CADTH is a not-for-profit organization that conducts HTA for Canada’s health care decision-makers. (CADTH is Canada’s drug and health technology agency. CADTH was an acronym of “Canadian Agency for Drugs and Technologies in Health”. However, in 2021, “CADTH” became the agency’s official name).

To inform the work that CADTH has done in this space, our information specialists conducted a limited literature search on key resources including MEDLINE, Embase, the Cochrane Methodology Register, the Health Technology Assessment database, the websites of Canadian and major international health technology agencies, as well as a focused internet search. The literature search strategy was an update of a strategy developed in 2018 for a previously published report [11]. It was comprised of both controlled vocabulary, such as the National Library of Medicine’s MeSH (Medical Subject Headings), and keywords. Our original main search concepts were environmental aspects/impact and health technology assessment. Database searches were run on 27 March 2019 to capture any articles published since 2018, search alerts were maintained until 20 February 2021. The search was run again on 10 June 2022, after which search alerts were maintained until 5 July 2022. The grey literature search, originally done in July 2017, was updated from 9–12 March 2021, then run again on 16 June 2022 to include the additional search concepts of environmental sustainability, pollution, carbon, and climate change. Search alerts were maintained until 5 July 2022. In addition, targeted consultations with key Canadian and International stakeholders were conducted between May 2019 and February 2021. Consultation contacts were identified by searching the grey literature, by CADTH Liaison Officers, through stakeholder suggestions, and through other available networks. Consultations were comprised mostly of health technology assessors, clinicians, environmental assessors, procurement specialists, academic researchers, governmental decision-makers, and associations involved in the provision of sustainable health care.

## 2. The Deliberative Process in HTA

Deliberation is a crucial part of HTA. This is when the strength and quality of the data—the clinical evidence, cost-effectiveness analysis, and any other factors of interest—are assessed, considered, and debated. Deliberation is carried out by an expert review committee comprising individuals with different experiences, perspectives, and values. A fulsome review of deliberative processes used by HTA committees to appraise the various types of data has been conducted by Richardson et al. [12].

In publications from the Health Technology Assessment International (HTAi) 2020 Global Policy Forum, the deliberative process was conceptualized using an input-throughput-output (ITO) model [13]. “Input” is the collection of evidence, information, and perspectives that forms the basis for deliberation. “Throughput” is the actual deliberation; that is, the presentation and weighing of facts, values, and reasons that leads to a collective judgement. “Output” is the stage at which the recommendation and the deliberation that preceded it are publicly posted for stakeholder feedback, which is then incorporated into the final recommendation [13]. The model also includes 3 core principles—transparency, inclusivity, and impartiality [13].

There are different ways environmental data can be gathered for use as inputs during deliberation. There are some environmental considerations that can be included as part of the economic evaluation of a health technology. In other cases, however, it would be more practical to consider environmental data separately, such as when the relevant environmental information or perspectives cannot practically be included in economic analyses or when the responsibility for determining the relative importance and weight of environmental impacts rests with decision-makers who need to take local context, priorities, and perspectives into account. It should be noted that, because the environmental aspects of health technologies are often not well studied, there may often be a lack of high-quality data to use as inputs.

To satisfy the core principle of transparency, the environmental data considered should be fully described, as well as how it was considered and how it factored into the decisions. This will allow policy-makers to determine if the deliberations and resulting recommendations are fair. As with any other factor assessed during an HTA, the limitations of the evidence should be considered during deliberations and clearly reported in resulting HTA recommendations.

Some HTA agencies have already included environmental considerations in their assessments, albeit on an ad hoc basis. CADTH has incorporated data on environmental issues associated with health technologies in only a small number of its assessments. An evaluation of community water fluoridation programs considered and included the amount of fluoride these programs release into water and soil [14]. An evaluation of dental amalgams and composite resins examined the contribution of mercury from dental fillings into the ecosystem [15]. National Institute for Health and Care Excellence (NICE) in the UK considers the reduction in GHG emissions [16,17] when evaluating anesthesia gas conserving systems.

For the CADTH assessments, the environmental data were gathered using a targeted literature search. In the review of community water fluoridation programs, the data were categorized according to the ecological receptor being exposed, the route and duration of exposure, and the hazard (or inherent toxicity) of the chemical—in this case, fluoride. A toxicological risk was considered possible if all 3 components were present [14]. In the dental amalgams and composite resins evaluation, relevant environmental data from the retrieved literature were categorized by hazard (e.g., what potentially toxic chemicals are present in the material—in this case, mercury), exposure (e.g., how might key receptors be exposed), and toxicology (e.g., what the potential toxic effects might be). In this evaluation, the data were also used to inform the economic review so that the annual cost in Canada of managing mercury waste from dental amalgams could be estimated [15].

The throughput—or the deliberative process—is likely the best way to assess the identified environmental considerations of a health technology alongside other key aspects appraised by the HTA, such as the clinical and cost-effectiveness of the technology. The deliberative framework [18] used by the Health Technology Expert Review Panel (HTERP) at CADTH already includes environmental impact considerations. The core members of HTERP include individuals with qualifications in evidence-based medicine and/or critical appraisal, an ethicist, a health economist, a health care practitioner, and a public member who represents the broad public interest. Depending on the technology being assessed, other specialists are added to provide specific additional subject matter expertise. During deliberation, panel members discuss their own experiences and insights on relevant environmental issues as detailed analyses on environmental issues are typically not included as part of the HTAs that guide their recommendations [10].

If environmental factors are to be assessed more regularly or frequently, it would be beneficial to include people with expertise in environmental considerations as core members on expert committees. This would help ensure that the principle of inclusivity is satisfied, that the values and perspectives of the people impacted by the recommendations are more fully represented, and that any resulting policy recommendations more accurately reflect the reality of the people who will be impacted. The potential conflicts of interest of these new members would have to be managed (as should be the case with all committee members) to satisfy the principle of impartiality and improve the credibility of the recommendations.

In addition, the deliberative process would benefit from having specific frameworks to guide deliberation on environmental issues and help members weigh these factors alongside the health benefits of a health technology and the factors from other domains. These frameworks could help committees ensure that as many potential environmental impacts of a technology as possible are captured during an assessment (e.g., a checklist of potential environmental impacts to consider).

The output of the deliberations, or recommendations, must convey how the inputs were obtained and how they were deliberated. This allows policy decision-makers to understand how the information regarding environmental considerations was integrated into the recommendations and what uncertainties remain [19].

## 3. When to Consider Environmental Considerations in HTA

Although it would be ideal to include environmental considerations for every health technology, at present this is not practical. This is both because the relevant data “inputs” for expert review committees to consider during deliberation are not always available, and because HTA agencies often lack the internal capacity to assess environmental factors for every health technology they assess. A triage process could help HTA producers choose the health technologies for which an assessment of environmental considerations would be most helpful and how extensive the assessment should be. This process should include a list of criteria that reflect the environmental impact a health technology could have.

Criteria that could trigger an assessment of environmental impact during an HTA may be present at any point across the product’s lifecycle—from its manufacture and distribution to its use and disposal. The criteria fall generally within three categories: whether there are toxic substances associated with the technology; whether the technology satisfies the principles of waste management; and the GHG emissions emitted by, or potentially reduced by, the technology [18].

Toxic substances: If a health technology is known to be produced using, contains, or emits toxic substances, this could trigger the consideration of environmental impacts. The Stockholm County Council list of chemicals hazardous to the environment and human health [20] was suggested by a number of stakeholders CADTH consulted as a useful resource, as was the Council’s list of environmentally classified pharmaceuticals [21]. These lists include substances deemed to be hazardous to the environmental due to the fact that they resist degradation, accumulate in the tissues of organisms, or are toxic to organisms [20,21]. Another trigger could be whether the health technology contains known endocrine disruptors that can leach into the environment (e.g., parabens, which are used as preservatives in pharmaceuticals).

Waste production and management: Concern about whether a health technology satisfies the waste management principles of refuse/reduce/reuse/repurpose/recycle could be another trigger. This could include technologies that are known to have a high environmental impact—such as frequently used disposable devices like inhalers, insulin injectors [22], disposable surgical custom packs [23,24], and medications packaged in larger quantities than needed by a single patient (which results in wasted product) [4]. It could also include technologies that may mitigate the environmental impact of health technologies (e.g., reusable or re-processible devices) [22].

GHG emissions: The manufacture and use of some health technologies can contribute a significant amount of GHGs. Therefore, knowledge that a technology produces a high level of GHGs could trigger an assessment of environmental considerations. Examples include inhaled anesthetics, robotic surgery [23,24], and pharmaceuticals [2]. Technologies that could reduce GHGs could also trigger an environmental review—for example, virtual health technologies that may reduce the need for people to travel to medical facilities [25] and IV anesthetics as an alternative to inhaled anesthetics [23]. Of all the possible triggers, this characteristic may be the easiest to get data on because methods and calculators to estimate GHGs have already been developed (e.g., the Care Pathway Carbon Calculator [https://shcpathways.org] (accessed on 26 July 2022)). Ways to assess other types of environmental impact are less developed.

As we previously mentioned, the availability of environmental data is currently limited and, as such, there will be challenges ahead in terms of determining who is responsible for collecting these data, ensuring it is of high quality, and making it available for HTA agencies to use.

## 4. Next Steps and Current Policies

Health journals from around the world recently released a joint statement calling for immediate action on climate change from governments, health professionals, and health systems [26]. In response, several HTA agencies have expressed their intention to launch new initiatives to reduce the contribution of health care to climate change [7,27,28,29]. They are asking for input from the public [7], exploring ways to incorporate environmental impact data into guidance development [27], identifying relevant research questions for researchers and methodologist to focus on [29], soliciting research evaluations of interventions or services to support more sustainable health care systems [30], and adapting HTA methodologies to allow the environmental impacts of a health technology to be assessed [19]. However, most HTA agencies do not yet consider, or they give limited consideration to, environmental impacts as part of their deliberative processes [10].

The World Health Organization (WHO) WHO-INTEGRATE evidence-to-decision framework is used by the WHO for guideline development and is recommended globally for health care decision-making processes. It suggests including environmental impact considerations within its societal implications criteria [31]. However, the framework was released in 2019, before the more recent interest in more often and more consistently addressing environmental consideration in HTA.

The European Network for Health Technology Assessment (EUnetHTA) had previously included environmental considerations under the broader “safety” domain of their HTA model [32]. However, in the network’s 2021 “A future model of HTA cooperation” white paper, the authors acknowledge that new methodological guidance will be needed to support the expansion of their HTA frameworks and allow environmental impact to be assessed [33].

In the UK, at the beginning of 2022, the National Institute for Health and Care Excellence (NICE) announced that it is aiming to help reduce the environmental impact of its recommendations by investigating whether environmental impact data can be incorporated into how it assesses health technologies [34]. This initiative builds on the UK government’s 2011 commitment to achieve net zero carbon emissions by 2050.

At around the same time, the National Institute for Health Research (NIHR), which funds research projects based on NICE recommendations, issued a call to its research programs to investigate how health care could be provided in a more sustainable way, in support of the UK government’s net zero goal [30]. NIHR has suggested potential areas of interest, such as reducing emissions associated with inhalers and anesthetic gases, finding effective alternatives to single-use technologies, and investigating the effectiveness of interventions that can be delivered or enhanced virtually [30].

In October 2021, the US Agency for Healthcare Research and Quality (AHRQ) announced that it will be using its data resources to understand how climate change impacts human health and health care delivery and will use this information to inform the agency’s strategies. AHRQ is also developing a measurement framework and guide to help inform health care sustainability measures and evidence-based, system-level interventions to reduce health care’s GHG emissions [7].

CADTH is, similarly, responding to the emerging needs. CADTH has already developed a protocol for tailoring HTAs that includes criteria to help determine when environmental considerations should be fully assessed. Among CADTH’s next steps will be to continue to develop its methodological processes by assessing its internal capacity and expertise and identifying where CADTH might need to solicit external support to help implement these new methods and processes. Having a standard taxonomy for and approach to incorporating environmental considerations in HTA will help improve the confidence decision-makers have in the conclusions and recommendations that come out of these assessments [10]. As CADTH has recently taken a life-cycle approach to HTA, CADTH may assess the environmental impact and other considerations of a health technology at different time points over its life cycle—at the research and development stage, after it has been used in the “real world,” and after it has been displaced by other innovations [35].

## 5. Conclusions and Recommendations

Cochrane, in an editorial about synthesizing evidence on climate change for use in health system decision-making, calls for the prioritization of HTA work on climate change [29]. The authors suggest expanding the definition of evidence to include, among other sources, the judgement of experts. We suggest that experts on environmental issues could sit at the deliberative table to discuss these issues alongside other relevant considerations. To do this effectively will require a more complex mix of perspectives and values than has previously been the case [13]. Therefore, HTA agencies must provide deliberation committee members with education and training to help them to debate issues they do not already have a background in. For example, HTA agencies should provide sessions on how to deliberate each of the various components an HTA—including not only environmental issues but also clinical effectiveness; cost-effectiveness; patient perspectives; and legal, ethical, social, implementation, and policy implications—as well as on the process of HTA itself.

HTA agencies should also work closely with relevant stakeholders to develop methods for synthesizing that information [29]. As a next step, health system decision-makers should be asked to provide input into what environmental considerations they think should be included in HTA. CADTH is currently planning a number of activities for engaging various health system stakeholders in this conversation.

In terms of how to integrate environmental considerations into HTA, we are in the very early days. However, this topic will continue to be a topic of interest globally. Given the urgent need to address the impact of climate change on human health, HTA agencies should work quickly to develop new processes. These processes could be trialed on health technologies that have been triaged for environmental assessment, such as those well known to have an environmental impact. The methods should be modified, refined, and standardized over time, with the goal of routinely assessing environmental considerations in every HTA. As health system decision-makers become increasingly aware of the environmental impacts of health care, HTA organizations can play an important role in supporting decisions that help mitigate the health harms of climate change before climate change reverses the health gains health technologies have, up until now, provided [5].
